# Rhodopsin gene evolution in early teleost fishes

**DOI:** 10.1371/journal.pone.0206918

**Published:** 2018-11-05

**Authors:** Jhen-Nien Chen, Sarah Samadi, Wei-Jen Chen

**Affiliations:** 1 Institute of Oceanography, National Taiwan University, Taipei, Taiwan; 2 Institute de Systématique, Évolution, Biodiversité (ISYEB), Muséum National d’Histoire Naturelle–CNRS, Sorbonne Université, EPHE, Paris, France; Laboratoire de Biologie du Développement de Villefranche-sur-Mer, FRANCE

## Abstract

Rhodopsin mediates an essential step in image capture and is tightly associated with visual adaptations of aquatic organisms, especially species that live in dim light environments (e.g., the deep sea). The *rh1* gene encoding rhodopsin was formerly considered a single-copy gene in genomes of vertebrates, but increasing exceptional cases have been found in teleost fish species. The main objective of this study was to determine to what extent the visual adaptation of teleosts might have been shaped by the duplication and loss of *rh1* genes. For that purpose, homologous *rh1*/*rh1*-like sequences in genomes of ray-finned fishes from a wide taxonomic range were explored using a PCR-based method, data mining of public genetic/genomic databases, and subsequent phylogenomic analyses of the retrieved sequences. We show that a second copy of the fish-specific intron-less *rh1* is present in the genomes of most anguillids (Elopomorpha), *Hiodon alosoides* (Osteoglossomorpha), and several clupeocephalan lineages. The phylogenetic analysis and comparisons of alternative scenarios for putative events of gene duplication and loss suggested that fish *rh1* was likely duplicated twice during the early evolutionary history of teleosts, with one event coinciding with the hypothesized fish-specific genome duplication and the other in the common ancestor of the Clupeocephala. After these gene duplication events, duplicated genes were maintained in several teleost lineages, whereas some were secondarily lost in specific lineages. Alternative evolutionary schemes of *rh1* and comparison with previous studies of gene evolution are also reviewed.

## Introduction

Rhodopsin is an opsin belonging to the G-protein-coupled receptor (GPCR) superfamily. In this superfamily, different opsins can be distinguished according to their Schiff base structure and to phylogenetic relationships of genes encoding opsins [1,2]. In vertebrates, visual opsin genes are often expressed in either retinal cone cells (i.e., cone opsin genes) or rod cells (i.e., the rhodopsin gene) [2]. They display diverse phenotypes with a maximum wavelength absorption (λ_*max*_) in the range of the light spectrum located at wavelength ranges of visible and ultraviolet light spectra [2]. Variations or adaptations in organismal spectral sensitivity may have arisen through structures of opsins and of retinal chromophore, gene duplications, and evolution of gene regulation [3–7]. Four different classes of cone opsin genes, including short-wavelength sensitive 1 (*sws1*) and 2 (*sws2*), medium- to long-wavelength-sensitive (*m/lws*), and mid-wavelength/green-sensitive genes (*rh2*), which correspond well with the absorption spectra of their encoding opsins, can be found in vertebrates. These opsins mediate an essential step of color discrimination especially for animals living in sufficient light environments (e.g., terrestrial habitats, coral reefs, and freshwater lakes) [8]. Rhodopsin (encoded by *rh1*) is mainly responsible for the perception of light or image capture, and contrary to cone opsins its function is sensitive to restricted dim-light environments such as, for example, the deep sea where the light spectrum in the water column is restricted to a narrow waveband of blue light (470~490 nm) or eventually fades to complete darkness at depths below 200 m [9,10]. As previously described in detail [6], rhodopsin is thus essential for aquatic vertebrates, especially for those teleost fishes living at great depths. They rely on this ability of image capture to find food and mates and maintain various interspecific and intraspecific associations that have a selective effect on their fitness [6]. Whereas various aspects of the molecular evolution of other visual opsin genes have been fruitfully investigated [e.g., 7,8,11–13], case studies of the rhodopsin gene are relatively rare [14–18].

Some early studies [14,19] suggested that the adaptation to dark or deep-water environments by vertebrate visual systems relied on the molecular evolution of the rhodopsin gene. The peak of λ_max_ in environments (e.g., water) is consistent with the λ_max_ of rhodopsin carried by its host [16]. A short-wavelength shift of λ_max_ (from the typical value of rhodopsin of 500 nm to ~490 nm) observed in rhodopsin of some deep-sea fishes, might have resulted from mutations of some key amino acids sites [20–22]. This hypothesis was tested by comparing amino acid sequences of rhodopsin from deep-sea fishes with others living in shallow waters, but with limited taxonomic sampling [14,16,21,23]. However, using a diverse set of spiny-rayed fishes living at different water depths, no simple relationship was observed between mutations at these amino acid sites and the spectral fit of the visual system of a fish to the light level where it lives [24]. Other physical or developmental mechanisms might more easily achieve this adaptation. For example, a fish can adjust its levels of rhodopsin expression (to achieve concordance of the λ_max_ for rhodopsin and water) [22,25,26] to adapt to its environment even with its rhodopsin has no expected mutations at targeted amino acid sites. In addition, mesopelagic fishes such as lanternfish can undergo great diurnal vertical migrations to adjust to their light needs [9,27].

Another mechanism of visual adaptation is duplication of the rhodopsin gene [6,12]. In fact, two paralogous rhodopsin genes with different λ_max_ values (resulting from an amino acid replacement) were reported in a few anguilliform fishes (from a conger eel, and Japanese and European eels) [19,26,28]. Expressions of these two genes in Japanese eels in different sexual maturation stages help them adapt to different environments (fresh water and deep sea) during their life cycle [26]. Besides these anguilliform fishes, two rhodopsin copies were subsequently found in zebrafish (Cypriniformes) [18,29], in pearleyes (Aulopiformes) [30], and more recetly in a few species of the Otocephala including the Cypriniformes [31,32], Characiformes [33], Siluriformes [34], and Clupeiformes [33]. A hypothetical senario explaining the “rise” of rhodopsin genes in those teleost fish genomes was often proposed to be the result of a single event of gene duplication that coincided with the fish-specific genome duplication (FSGD), that occurred in the common ancestor of teleost fishes [17,18,33,35–40]. However, the hypothesis has not consistently been tested by an explicit phylogenetic method.

In this study, we attempted to provide a thorough perspective of rhodopsin gene evolution with an emphasis on early teleost fishes. We thus explored, using a polymerase chain reaction (PCR)-based method, public genetic/genomic data mining, and subsequent phylogenomic analyses, the presence of additional homologous rhodopsin genes in vertebrate genomes, including the non-visual exo-rhodopsin gene (extra-ocular rhodopsin, *exo-rh1*) that is expressed in the pineal gland of the fish brain [41]. Our taxonomic sampling focused on the Elopomorpha (tarpons, bonefishes, eels, and relatives) which is one of the three major extant teleost lineages, and includes more than 1000 species [6,42]. The morphology, ecology, and life history of elopomorph fishes vary widely. Most elopomorphs are marine fishes; they are bathypelagic or bathydemersal, and some live in shallow reefs (i.e., moray eels and snake eels). Only the fishes from the family Anguillidae spend part of their life in fresh water (catadromous life cycle). In addition, the consensus view reached by multiple recent nuclear gene studies shows that the Elopomorpha is sister to the rest of the teleosts [43–46]. Thus, the common ancestor of the Elopomorpha rose close to the divergence of teleosts, at a time which coincides with the FSGD event. The high diversity (in morphology, ecology, and behavior) and the phylogenetic position among elopomorph fishes (as a sister group to the rest of the teleosts) make this group of fishes an ideal model to test the hypothesis of the “rise” of rhodopsin genes in teleost fish genomes and address the role of gene duplication in the adaptation of visual systems of deep-sea teleost fishes [6].

## Materials and methods

### Ethics statement

This research was performed at National Taiwan University (NTU) in accordance with NTU’s guidelines regarding animal research. As this project did not involve experiments on live fish, no ethics statement was required. Most of the specimens examined in the present study were purchased from local fish markets or fish landing sites (Da-Shi and Donggang) in Taiwan; others were from museum specimens collected during exploratory cruises (campaigns: EXBODI, PAPUA NIUGINI, Taiwan 2013, NanHai 2014, and ZhongSha 2015) conducted between 2012 and 2015 under the "Tropical Deep-Sea Benthos" program and its joint bilateral cooperation research project entitled “Taiwan France Marine Diversity Exploration and Evolution of Deep-Sea Fauna” (TFDeepEvo) with the French research vessel *ALIS* and the Taiwanese research vessels *OR1* and *OR5* (S2 Table). A few samples were provided by our collaborators (see details in “Acknowledgments” and S2 Table).

### Sequence acquisition and data collection

Genomic DNA was extracted from a small piece of muscle tissue or fin cut from each examined specimen using a commercial DNA extraction kit (DNeasy Blood & Tissue Kit, Qiagen, Hilden, Germany) and/or LabTurbo DNA Mini Kit LGD480-220 (TAIGEN Bioscience, Taipei, Taiwan) following the manufacturer's protocols. With PCR methods, fragments of the rhodopsin gene were amplified by standard primers published in a previous study [47] or by modified or specific primers to the putative “deep-sea” type of rhodopsin gene homologous to those possessed by Japanese and European eels, to osteoglossomorph *rh1-1* and *rh1-2* of Goldeye (*Hiodon alosoides*) or to *rh1-B* of otocephalan fishes (see S1 Table). Temperature cycling profiles for amplification consisted of an initial denaturation stage (95°C, 5 min) followed by 35 cycles, each with a denaturation step (95°C, 40 s), an annealing step (54°C, 40 s), and an elongation step (72°C, 60 s), before a final extension stage (72°C, 7 min). PCR products were purified using the AMPure magnetic bead cleanup protocol (Agencourt Bioscience, USA). Purified PCR products were sequenced by Sanger sequencing using dye-labeled terminators. Sequence determinations from Sanger reaction products were generated on ABI 3730 analyzers (Applied Biosystems, Foster City, CA, USA) at Genomics BioSci & Tech (Taipei, Taiwan) and at the Center of Biotechnology (NTU, Taipei, Taiwan). Sequences newly reported in this study were deposited in GenBank under accession numbers: MH674300, MH769447~MH769543 (S2 Table).

Reference or compared sequences were first obtained by searching public genomic databases such as GenBank [48] and Ensembl [49] with key words like “*rh1*” or “rhodopsin”. To further explore for potential homologous *rh1-* or *rh1*-like sequences (e.g., *exo-rh1*) in jawed vertebrates, different runs of “BLAST” searches (function: blastn) were performed using known *rh1* (or *exo-rh1*) sequences such as freshwater/deep-sea-type rhodopsin sequences from the anguillids as the query sequence with default settings against sequences deposited in the Ensembl and NCBI (nucleotide collection) databases. Eventually, 227 rhodopsin gene homologous sequences, including *exo-rh1*, intron-containing *rh1*, and intron-less *rh1*, from 179 vertebrate species were included in this study (S2 Table).

### Sequence alignment and data matrix

Intron regions of rhodopsin sequences were removed (except from *rh1* sequences from ray-finned fishes which have no introns), terminal ends were trimmed, and remaining parts of the sequences were manually aligned based on the inferred amino acid translation using Se-Al vers. 2.0a11 [50] before the phylogenetic analyses. The final alignment contained 996 nucleotides. We characterized the base composition and tested for significant deviation from base composition homogeneity (by codon position) using a Chi-squared test as implemented in PAUP* vers. 4.0a10 [51] (results are showed in S3 Table). To reduce the impact of homoplasy due especially to base composition bias at the third codon position sites on phylogenetic estimates, we used an RY-coding strategy by recoding “A” and “G” as “R”, and “C” and “T” as “Y” at the third codon positions when constructing the data matrix [52,53] using MacClade [54].

### Phylogenetic analysis

The compiled data matrix with 227 rhodopsin gene homologous sequences was applied to infer the *rh1/rh1-like* gene tree using the maximum-likelihood (ML) method. Sequences from a shark (*Scyliorhinus canicula*) and skate (*Raja erinacea*) were used as outgroups to root the inferred tree. For the ML search, five independent runs were conducted using the GTR + G model as implemented in RAxML [55], and the final ML tree was selected among the five best trees of those runs. Nodal support was assessed by bootstrapping [56] based on 1000 pseudo-replicates generated from five separate runs. All RAxML analyses including bootstrapping were conducted on high-performance parallel computers accessed using the CIPRES Science Gateway vers. 3.3 at http://www.phylo.org [57].

### Hypothesis evaluation

**Fig 1:**
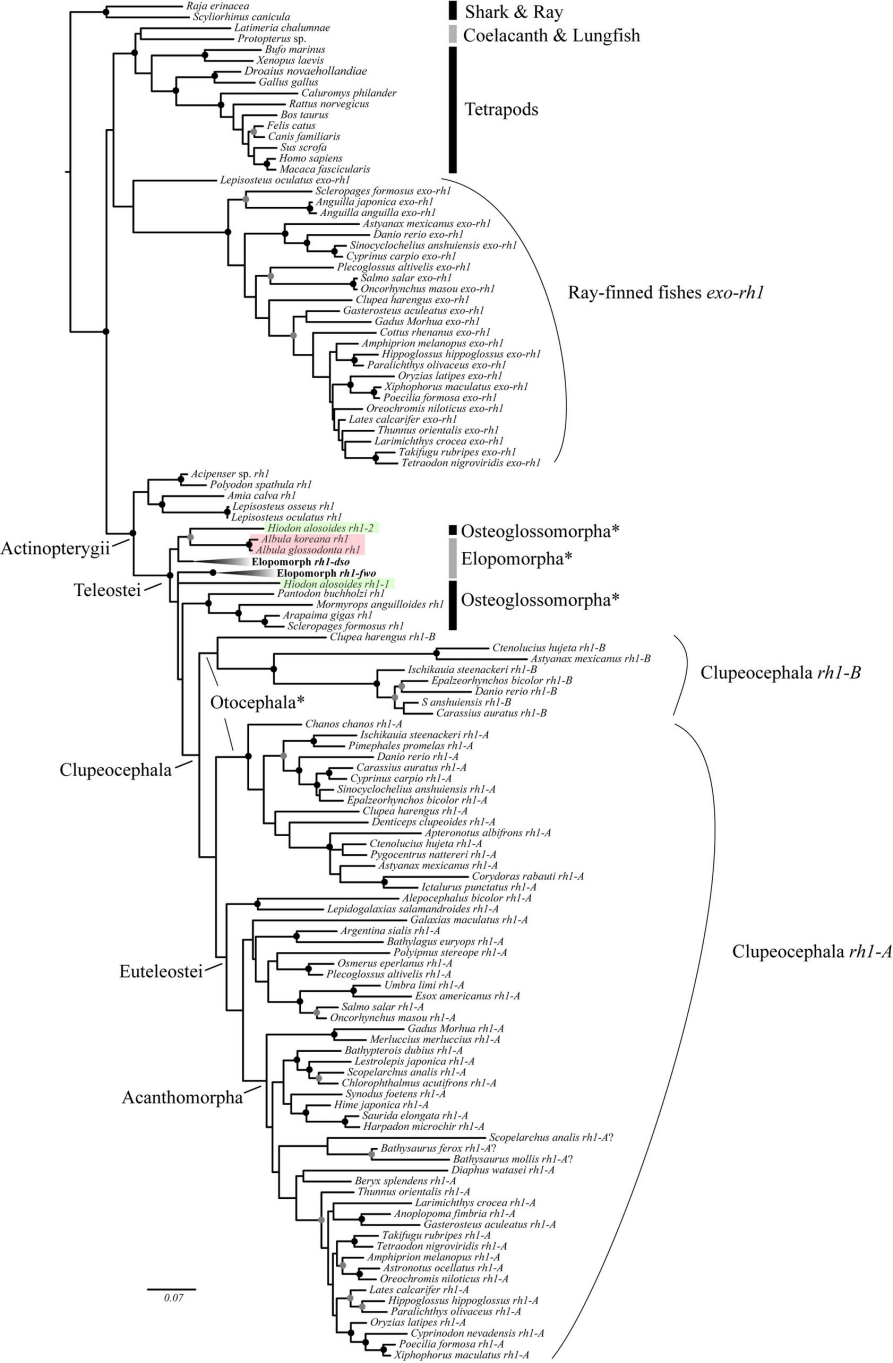
The *rh1* gene tree of jawed vertebrates reconstructed using ML method. Circles on the nodes represent the different degrees of nodal supports in terms of the bootstrap values from ML analysis (above 79%, black; 60–79%, gray). Bootstrap values below 60% are not shown. Asterisks indicate the non-monophyletic groups due to the result of gene duplications. Highlighted lineages were gene sequences with uncertain orthology and were further applied to the gene orthology assessment (see content, S1 Fig).

The phylogenetic analysis revealed some sequences with an uncertain orthology within the Teleostei. For those sequences, alternative orthology hypotheses were compared (see Fig 1 and S1 Fig). For example, the lineage *Albula rh1* (Elopomorpha: Albulidae) was forced to independently group with each elopomorph orthologous lineage, and the ML values of the constrained topologies were compared: the best likelihood tree (or hypothesis) was chosen. In the case of *Albula rh1*, its gene orthology to Elopomorph *rh1-dso* was more likely than to Elopomorph *rh1-fwo* (S1 Fig). Hypothesized topologies were constructed using Mesquite [58] and their ML values were calculated using RAxML.

**Fig 2:**
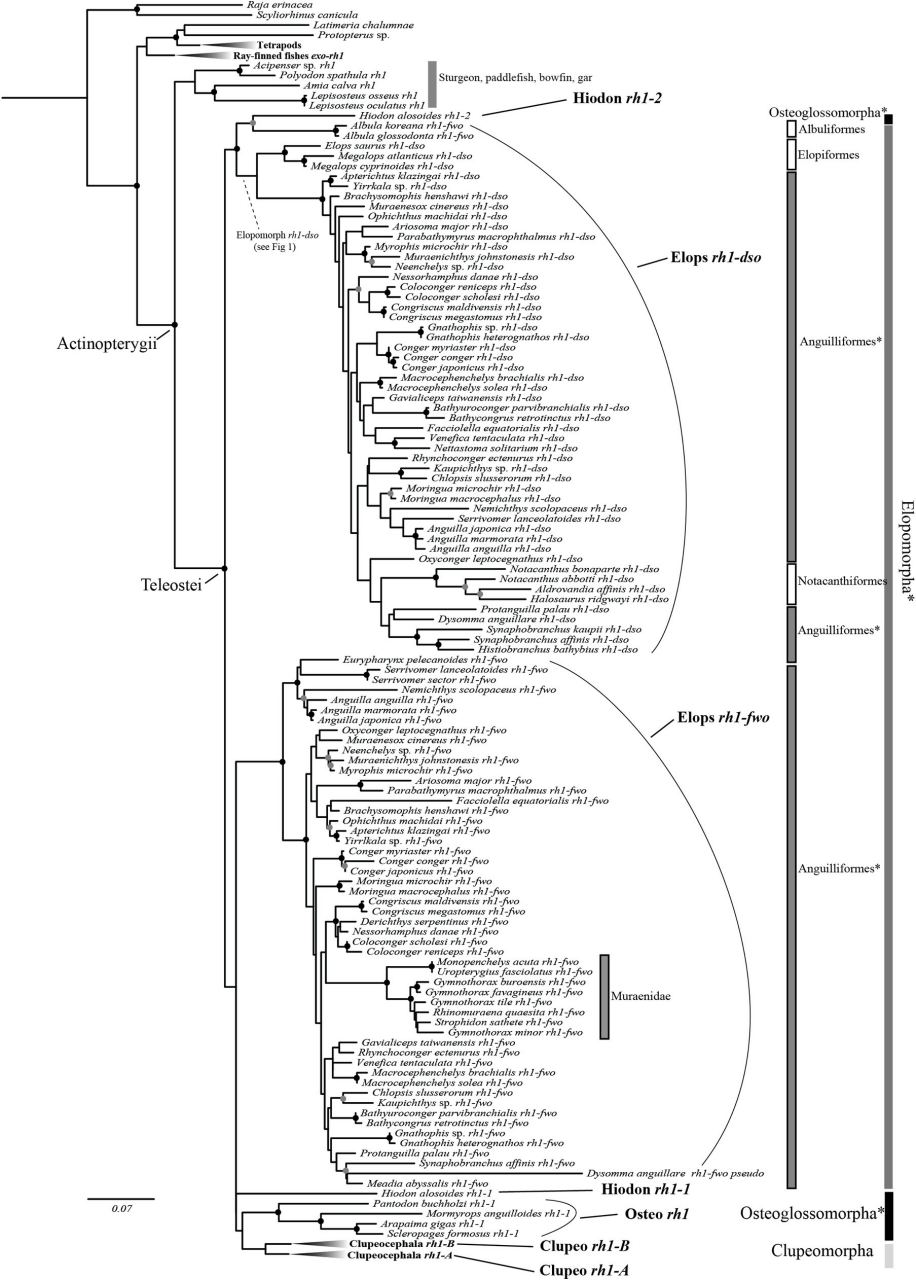
The *rh1* gene tree of jawed vertebrates reconstructed using ML method (with detail view of the elopomorph lineages shown). Circles on the nodes represent the different degrees of nodal supports in terms of the bootstrap values from ML analysis (above 79%, black; 60–79%, gray). Bootstrap values below 60% are not shown. Asterisks indicate the non-monophyletic groups. Annotations of abbreviations (i.e. Hiodon *rh1-2*, Elops *rh1-dso*, etc.) are corresponding to the lineages used in the hypothesis evaluation (Fig 3).

**Fig 3:**
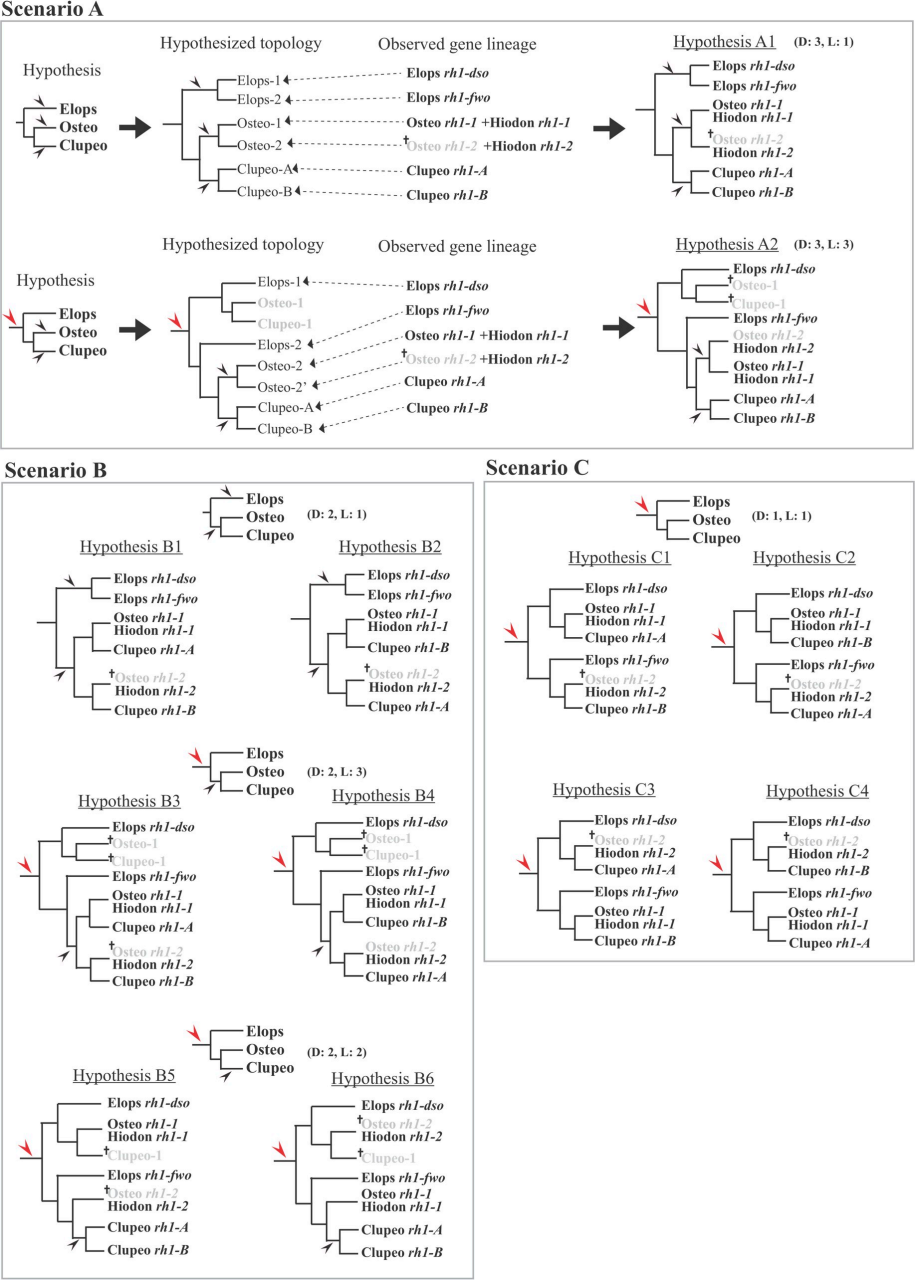
Alternative hypotheses of *rh1* gene evolution in the Teleostei. Scenario A, B, and C imply that the major gene duplication events occurred three times, twice, and once, respectively. Observed gene lineages are defined according to the *rh1* gene tree (see the annotation in Fig 2). Arrow sign indicates a gene duplication event (red arrow indicates the duplication that coincides with the FSGD event). Cross sign indicates a gene loss event. The event time(s) are showed behind each hypothesis: D, time(s) of gene duplication; L, time(s) of gene loss. Abbreviation: Elops, Elopomorpha; Osteo, Osteoglossomorpha; Clupeo, Clupeocephala.

Depending on the frequency of gene duplication events, based on results of the phylogenetic analysis (Figs 1 and 2) and assessments of the orthology (S1 Fig), three main hypothesized scenarios concerning *rh1* evolution in the Teleostei were proposed (Fig 3). In scenario A, gene duplications occurred three times during the evolutionary history of the Teleostei, either in each common ancestor of the main teleost lineages (Elopomorpha, Osteoglossomorpha, and Clupeocephala) (hypothesis A1, Fig 3) or one of the duplication events occurred before the divergence of the Teleostei (instead of in the common ancestor of the Elopomorpha) (hypothesis A2, Fig 3). In scenario B, gene duplications occurred twice, one in the common ancestor of the Osteoglossomorpha and Clupeocephala and the other either before divergence of the Elopomorpha (Fig 3, hypotheses B1 and B2) or before divergence of the Teleostei (Fig 3, hypotheses B3 and B4). Another alternative hypothesis in scenario B was that two gene duplication events occurred, one in the common ancestor of the Teleostei, and the other in the common ancestor of the Clupeocephala (Fig 3, hypotheses B5 and B6). Scenario C assumed that both copies of *rh1* found in the genomes of the Teleostei resulted from a single gene duplication event that coincided with the FSGD event (Fig 3, hypotheses C1~C4). All hypothesized topologies were subject to evaluation using the ML criterion. The constrained topologies that fit the alternative hypotheses were first constructed using Mesquite [56], and the respective RAxML analyses were performed to obtain their ML values for comparison and for further likelihood ratio tests using the AU-test as implemented in the computer program CONCEL [59].

### Ancestral state reconstruction (ACR)

In addition to the phylogenetic method, we also used ACR to investigate rhodopsin gene evolution. Here, the presence/absence of *exo-rh1*, intron-containing/intron-less *rh1*, and the copy number of intron-less *rh1* were used as independent characters. Ancestral states of these characters were inferred from a simplified phylogeny of jawed vertebrates based on several molecular studies [43,44,46,60,61] using Mesquite [58]. A parsimonious approach was applied since it allows missing data and generates ancestral states that minimize the number of evolutionary steps.

## Results

### Rhodopsin gene sequences and phylogenetic tree

In total, 98 newly generated rhodopsin gene homologous sequences from the Teleostei (93 from the Elopomorpha, two from the Osteoglossomorpha and three from the Clupeocephala) and 129 *rh1/rh1-like* sequences retrieved from the literature and databanks were included in the analysis (S2 Table). Our gathered homologous sequences were sampled from a wide taxonomic range of jawed vertebrates with dense sampling within the basal-most lineage of the Tetelostei, the Elopomorpha (Figs 1 and 2). Sequences of the Osteoglossomorpha were from five different species, including *Hiodon alosoides*, which belongs to the basal-most osteoglossomorph family. With this sampling strategy, analytical results should have allowed us to appropriately interpret *rh1* gene evolution of jawed vertebrates.

The inferred *rh1* gene tree was roughly consistent with the species phylogeny of jawed vertebrates with some exceptions probably resulting from limitations or artifacts of the phylogenetic reconstruction using sequences from a relatively short fragment of a single gene (Fig 1). For instance, *exo-rh1* orthologous sequences found in genomes of ray-finned fishes formed a monophyletic group that might be sister to the tetrapod rhodopsin clade [with a bootstrap value (BP) of < 60%] rather than to the ray-finned fish-specific rhodopsin clade, which was unexpected (Fig 1). A previous study hypothesized that the duplication event from which *exo-rh* arose occurred in the common ancestor of all ray-finned fishes, excluding the bichirs (the Polypteriformes) [41]. Unfortunately, no rhodopsin homologous sequences were available for bichirs to test this hypothesis. Nonetheless, the ancestral state reconstruction more or less supported the hypothesis proposed by Mano et al. [41] (see results below).

**Fig 4:**
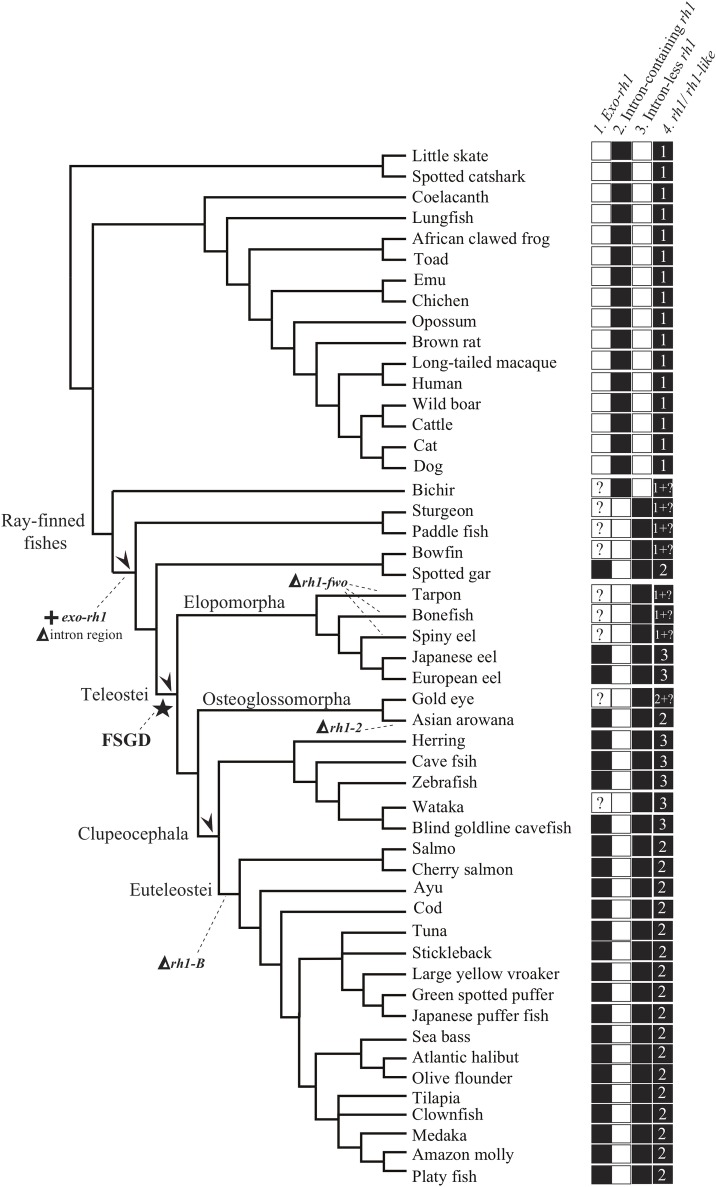
The summary of the rhodopsin gene features and its evolution within jawed vertebrate. Panels behind the tree represent status of different characters of the gene. Blank squares indicate that the character is absent while solid ones indicate the character present in the genome of the organism. Question mark indicates that the status of the *exo-rh1* gene is unknown from the genome of the organism. The numbers within the solid squares in the third panel show the copy number of intron-less *rh1* found in the genome of the organism. The last panel indicates the number of *rh1* and *rh1*-like (i.e. *exo-rh1*) gene can be found in the genome of the indicated organism. Arrows indicate the gene duplication events and plus marks show the duplicated gene, triangles indicate the intron region deletion and the gene loss events. FSGD, Fish-Specific Genome Duplication event.

The ray-finned fish-specific rhodopsin gene, which is expressed in retinal rod cells, is an intron-less gene [41,62]. None of our obtained sequences of teleost *rh1* in this study contained introns, which further supports this hypothesis. In the inferred tree, all of the compiled sequences of ray-finned fish *rh1* formed a highly supported monophyletic group, in which sequences of the Teleostei formed another strongly supported clade sister to the non-supported clade containing sturgeon, paddlefish, bowfin, and gar (Fig 1). Within the Teleostei clade, four main (but relatively weaker supported) groups/lineages could be found: (i) the *Hiodon alosoides rh1-2* and *Albula* spp. *rh1* plus all sequences attributed to Elopomorph *rh1-dso*; (ii) all the elopomorph *rh1-fwo* sequences; (iii) *Hiodon alosoides rh1-1*; and (iv) all osteoglossomorph *rh1* sequences excluding the *Hiodon alosoides rh1-1* and all clupeocephalan *rh1* sequences (Fig 1). Clupeocephalan *rh1* can be subdivided into two reciprocal monophyletic groups, Clupeocephala *rh1-A* and Clupeocephala *rh1-B*, which indicates a putative gene duplication event might have occurred in the common ancestor of clupeocephalan fishes (Fig 1). For a summary in terms of copy numbers of *rh1/rh1-like* genes, after our phylogenetic assessment, we concluded that whereas only one copy (intron-containing *rh1* gene) is present in genomes of the skate, shark, coelacanth, lungfish, and tetrapods, up to three copies were detected in genomes of diploid ray-finned fishes (two intron-less *rh1* genes and one *exo-rh1* gene) (S2 Table; Fig 4). The two copies of the intron-less *rh1* gene were present in all three main teleost groups, i.e., two copies (*rh1-dso* and *rh1-fwo*) were found in genomes of many elopomorph species (most of the anguilliforms); two copies (*rh1-1* and *rh1-2*) were found in the genome of *Hiodon alosoides* (Osteoglossomorpha); and both the *rh1-A* and *rh1-B* genes were found in genomes of some clupeocephalan species from the Otocephala (Fig 1).

### Gene orthology and hypotheses of gene evolution

To resolve the uncertainty of *rh1* gene evolution within the Teleostei inferred from the reconstructed gene tree, we assessed the gene orthology and evaluated alternative hypotheses of genes using the constrained analysis described in the "Methods" section.

Considering the best tree score (-ln likelihood) among the constrained trees corresponding to alternative hypotheses, we suggest that *Hiodon alosoides rh1-1* is most likely related to the other osteoglossomorphs *rh1* sequences, and *Hiodon alosoides rh1-2* is a paralog of *Hiodon alosoides rh1-1*. In reference to these results for the two copies of *Hiodon alosoides*, other Osteoglossomorpha *rh1* sequences are thereafter considered as belonging to the “*rh1-1”* group. We also considered that sequences from single copies of *rh1* found in genomes of *Albula* spp. are most likely related to Elopomorph *rh1-dso* rather than to Elopomorph *rh1-fwo* (see S1 Fig). The lineages we used to evaluate the hypothesis were defined based on the *rh1* gene tree and results of the gene orthology assessment (Fig 2 and S1 Fig). Subsequently, according to the ML estimation (Table 1), the best -ln likelihood score hypothesis B6 should be chosen to represent the most likely scenario for *rh1* gene evolution in the Teleostei (Fig 3). After further evaluation with likelihood ratio tests, we found that although the other alternative hypotheses (except B2) in addition to B6 could not be excluded to explain the results of gene rises and falls of the *rh1* gene in teleost genomes by *p*-values resulting from the AU-test, with a non-scaled probability (np), all hypotheses except hypotheses B5 and B6 were rejected (Table 1). Therefore, by following the most likely hypothesis (B6) selected, the *rh1* gene likely should have been duplicated twice (instead of once) during the early evolution of the Telesotei: one occurred before divergence of the Teleostei, and the other occurred in the common ancestor of the Clupeocephala (Fig 3). Yet, the duplicated *rh1* from the first duplication event within the Teleostei was apparently lost or simply was not found (due to PCR failure or incomplete data) from the available genomic data of several lineages in the Osteoglossomorpha (except *Hiodon alosoides*) and in the Clupeocephala (hypothesis B6, Fig 3).

**Table 1:** Likelihood value and probability of the best tree that inferred under the hypothesized constraint of each hypothesis concerning the gene evolution (see Fig 3). The *p*-value shown in bold indicates that the hypothesis could not be rejected.

	- ln likelihood			
Best tree	-33958.863478			
Hypothesis		-ln likelihood Ranking	au	np
A1	-33982.52599	6	**0.137**	0.013
A2	-33977.39867	3	**0.152**	0.012
B1	-33990.4781	11	**0.104**	0.016
B2	-33987.99438	9	0.043	0.003
B3	-33985.72485	7	**0.095**	0.004
B4	-33980.32094	4	**0.166**	0.011
B5	-33975.71097	2	**0.269**	**0.075**
B6	-33960.44312	1	**0.687**	**0.367**
C1	-33993.89872	12	**0.083**	0.020
C2	-33987.38234	8	**0.208**	0.019
C3	-33981.15311	5	**0.184**	0.024
C4	-33989.47416	10	**0.08**	0.002

### Ancestral state reconstruction (ASR)

ASR suggests that *exo-rh1* might have resulted from a duplication event that occurred either before the divergence of the ray-finned fishes or just after their divergence with the bichir (S2 Fig). Deletion of *rh1* intron regions occurred after divergence of ray-finned fishes with the bichir (S2 Fig). These two events might have occurred simultaneously. Based on the ASR analysis, three independent events (instead of one) of gene duplications of *rh1* intron-less genes likely occurred within the Teleostei (S2 Fig).

## Discussion

### Exo-rhodopsin in ray-finned fishes

The *exo-rhodopsin* of ray-finned fishes was first discovered by an early study investigating gene expressions in the photosensitive pineal gland of zebrafish [41]. The *exo-rhodopsin* gene is thought to have the same role as other non-visual opsin genes (like *pinopsin*, *parapinopsin*, etc.) that are expressed in the pineal gland and regulate the rhythmic production of melatonin and thereby regulate circadian rhythms [41,63,64]. While *rh1* genes found in ray-finned fishes are intron-less, the structure of pineal *exo-rh1* with five exons and four introns is similar to the rhodopsin gene of the other vertebrates. It is suggested that the intron-less *rh1* may have arisen through an ancient retrotransposition of mature mRNA originating from *exo-rh1*. The duplication event, resulting from this retro-duplication mechanism, occurred early in the evolution of ray-finned fishes since *rh1* of the sister-clade of the Teleostei (i.e., sturgeon, bowfin, and gar) is also intron-less [65,66]. With increasing available genomic data, more and more *exo-rh1* genes have been identified from genomes of diverse ray-finned fishes [33]. In this study, we present 27 *exo-rh1* sequences which were found in spotted gar (*Lepisosteus oculatus*), two anguillids (Elopomorpha), *Scleropages formosus* (Ostoglossomorpha), and several species of the Clupeocephala, notably from model species from which complete genomic sequences are available (i.e., *Danio rerio*, *Oryzias latipes*, *Takifugu rubripes*, etc.) [41,61,67,68] (Fig 1). The presence of *exo-rh1* throughout this wide taxonomic coverage of ray-finned fishes highlights its functional importance in the evolution of ray-finned fishes [69].

### Gene duplication in the teleostei

Teleost fishes are usually found to contain more copies of genes (e.g., opsin genes) than other vertebrates; this might be a result of the genome-wide duplication events that occurred during their evolution [39]. In addition to the two rounds of whole-genome duplication (WGD) events that occurred at the common ancestor of vertebrates, teleost fishes experienced a third round of WGD (the FSGD event) which occurred in their common ancestor [36–40]. While most of duplicated genes are lost (nonfunctionalization) [40], those that are retained are assumed to be maintained either by a proportioning of ancestral gene functions (subfunctionalization) or by their evolution into novel functions (neofunctionalization) [70]. These various evolutionary dynamics of gene evolution might explain the great diversity of teleost species [3,71–74]. As a consequence of this specific event of ancient genome duplication (i.e., the FSGD), the Teleostei are a good model group to investigate how gene duplications or large-scale genomic changes can shape the biodiversity of a lineage [3,36,75].

Phylogenetic analyses in previous studies were conducted to address ray-finned fish specific intron-less *rh1* gene evolution, but conflicting results were obtained. To figure out the origin of the second visual rhodopsin gene (paralogous *rh1*) found in zebrafish and other ray-finned fishes (cavefish and carp), Morrow et al. [18] analyzed around 130 *rh1*/*rh1*-like sequences, and their taxonomic sampling expanded from lampreys throughout the vertebrates (see Fig 4 in [18]). The topology of the gene tree from Morrow et al. [18] is similar to the gene tree obtained in this study (Fig 1), except for the position of the paralogous *rh1* clade which was closely related to anchovies, herrings, and ostariophysians (see Fig 4 in [18]). Those authors therefore rejected the lineage-specific duplication hypothesis and suggested a much more ancient origin that linked to FSGD for the second visual rhodopsin gene found in zebrafish [18]. Yet, no further hypotheses for *rh1* evolution were proposed or tested in Morrow et al. [18]. Recently, *rh1* evolution (duplication) within the ray-finned fishes was further studied with whole-genomic sequences. Lin et al. [33] identified visual opsin genes and their adjacent genes (sytenenies) from 59 ray-finned fish genomes, and restated a model of rhodopsin gene evolution. Largely based on syntenic data, they found that *rh1* duplicates was retained after whole-genome duplication in the ancestor of teleosts (FSGD), and indicated that paralogs found in eels and zebrafish both originated in a single gene duplication event [33]. However, in their *rh1* gene tree (S3 Fig), it is more likely that the lineage-specific duplication events occurred in eel and herring lineages (S3 Fig). This topology barely supported the hypothesis and conflicted with their conclusion [33]. A similar hypothesis (single gene duplication) for *rh1* evolution was also reviewed by Nakamura et al. [17]. Nakamura et al. [17] compared syntenic structures along *rh1* genes found in spotted gar, Japanese eel, Asian arowana, and other representative teleosts in detail and deduced gene duplication/loss events under different scenarios. They proposed that *rh1* duplicates which were products of the FSGD had been maintained in the genomes of Japanese eel and some otocephalan species (i.e., zebrafish), but one of the duplicated copies was lost in the genomes of arowana and the Euteleostei (i.e., Nile tilapia) (see Fig 5 in [17]). According to the synteny, the authors further suggested that losses of *rh1* on opposite regions occurred independently in the arowana and clupeocephalan lineages after their divergence (see Fig 5 in [17]). Although this hypothesis (single gene duplication) was strongly supported by their phylogenetic analytical results of concatenated sequences [*rh1* plus adjacent genes (*ataxin7*, *magi1*, *prickle2*)], the topologies of some individual gene trees (i.e., *ataxin7*, *rh1*, and *magi1*) failed to support this hypothesis [17]. The authors herein simply concluded that two copies of the eel rhodopsin gene were most likely generated at the FSGD which actually corroborated our result (hypothesis B6 in Fig 3), and less stressed the story of two copies found in other teleosts like zebrafish. Adjacent genes along with *rh1* were also analyzed by Lagman et al. [75], in which topologies from those genes were inconsistent (not showed in the text). The authors therefore could not ascertain whether or not teleost *rh1* paralogs (in anguillid eels, zebrafish, and cyprinids) originated from the FSGD [75].

Since the two copies of the ray-finned fish specific *rh1* gene were present in all three main teleost groups, the most straightforward answer for the question of the rise of those paralogs in teleost genomes is that gene duplication events independently occurred in the specific lineages (Fig 3, scenario A; S2 Fig). Yet, this is not necessarily the most parsimonious solution, like the above studies which demonstrated scenario C in Fig 3 that involves a single duplication before teleost diversification. Such a question should be addressed by an explicit phylogenetic method as presented in this study. Multiple hypotheses concerning various *rh1* evolutionary events were subsequently tested. The likelihood score comparison and results of the test showed that hypothesis B6 was the most likely interpretation of *rh1* gene evolution in the Teleostei (Table 1; Fig 3, hypothesis B6). It is suggested that the *rh1* gene was duplicated once before the explosive divergence of the teleosts, which might correspond to the whole-genome duplication event of the FSGD (Fig 4). Following this gene duplication, the duplicated *rh1* was maintained in elopomorph fishes (Elops *rh1-dso* and *fwo*) and *Hiodon alosoides* (Osteoglossomorpha) (Hiodon *rh1-1* and *rh1-2*) but secondarily lost twice in other osteoglossomorph species and the Clupeocephala (Fig 3, hypothesis B6; Fig 4). Subsequently, one more duplication event supposedly occurred in the common ancestor of the Clupeocephala, which gave rise to the second copy of *rh1* found in the Clupeocephala (Clupeocephala *rh1-B*) (Fig 3, hypothesis B6). According to the phylogenetic results and a thorough survey of possible genes that are orthologous to each of the identified teleost rhodopsin genes through lab work and data mining of the whole genome (Ensembl) and NCBI Genbank databases, it was observed that after gene duplications, gene loss events regularly occurred. For example, several elopomorph fishes like tarpons (Elopiformes), bony fishes (Albuliformes), and spiny eels (Notacanthiformes) lost their *rh1-fwo* (Figs 2 and 4). The inferred secondary loss of one of the Clupeocephala genes (*rh1-B*) during the early evolution of the Euteleostei (Figs 1 and 4) was supported by the orthologous gene search of *rh1-B* using BLAST against available complete genomic sequences of model euteleost species deposited in the Ensembl database [49].

**Fig 5:**
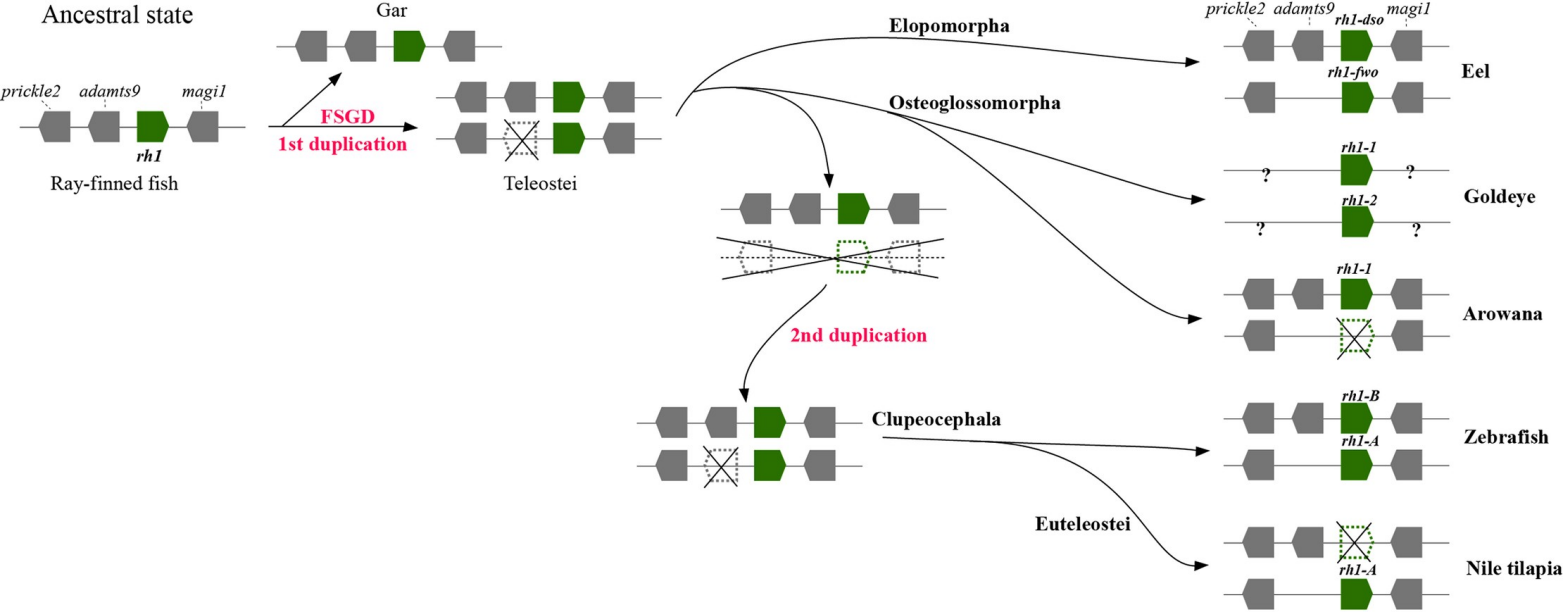
Hypothesized evolutionary scheme of *rh1* region in teleosts. Two times of gene duplication events were demonstrated. Furthermore, it is suggested that one gene cluster was lost before the second gene duplication event occurred in the common ancestor of the Cupeocephala. Rhodopsin gene is showed in green color while the adjacent genes in grey. Crosses indicate the cluster/gene lost event.

The major difference in the proposed hypotheses for *rh1* evolution in ray-finned fishes between the present and previous studies is the second duplication event which supposedly occurred in the common ancestor of the Clupeocephala. It is likely that the extra *rh1* found in clupeocephalan lineages like zebrafish, herring, etc. resulted from this event rather than the first duplication event (the FSGD). This hypothesis was also supported when referring to the gene tree of *magi1* (with an RY-coding strategy) from Nakamura et al. [17] (showed in their supplementary data) and the *rh1* gene tree from Morrow et al. [18], in which the sequences from both paralogous gene lineages of the Clupeocephala formed a monophyletic group. Combining the hypothesis proposed in this study with whole-genomic data (synteny) from references [17,33], an evolutionary scheme of the *rh1* regions in teleosts is presented in Fig 5.

In addition, it was noted that the topologies of the *rh1* tree with the best likelihood scores from previous references mentioned above as well as from this study (Figs 1 and 2) all separated the elopomorph *rh1* paralogs to be non-monophyletic or paraphyletic. This pattern can be explained by the FSGD hypothesis, since the two *rh1* genes found in elopomorphs were products of this duplication event, which occurred before teleost divergence. A pattern of reciprocal sister-group relationship of the two elopomorph paralogs would be observed if only a lineage-specific duplication event had occurred before the divergence of the Elopomorpha (see: hypotheses A1, B1 and B2 in Fig 3).

This study, however, had several limitations. For example, without the whole-genomic sequences, it could not be determined whether the absence of the other *rh1* in some elopomorph lineages like tarpon, bonefishes, and spiny eels (Fig 4) was due to a gene loss event or experimental failure (PCR-based strategy). Also, the length of *rh1* might be too short to provide sufficient information for phylogenetic inferences, which might result in a tree topology with low support at nodes that represent long-standing amphibious relationships of organisms. However, compared to the references based on whole-genomic data [17,33], our taxonomic sampling was expanded throughout jawed vertebrates, which allowed us to comprehensively test the hypothesis. Moreover, by examining the Fig 5 of this study, the syntenic structure could not reflect the true story of gene evolution, and the actual gene evolution might have been misrepresented (Fig 5) (see Results in [33]). To reduce biases as much as possible caused by limitations mentioned above, supplementary analyses were applied like ancestral state reconstruction (S2 Fig) and hypothesis evaluation (Table 1; Fig 3) in this study. Eventually, further data and research are needed to precisely address the gene evolution in ray-finned fishes such as whole-genomic sequences from early-teleost fishes like elopomorphs (i.e., tarpons, bony fishes, and spiny eels) as well as osteoglossomorphs (i.e. *Hiodon alosoides* to complete the scenario of gene evolution with syntenic structure as presented in Fig 5), since the genomes of those lineages represent missing puzzles between the early ray-finned fishes and the remaining teleosts to address questions concerning gene duplication.

### Two copies of the rhodopsin gene in the Elopomorpha

Anguillid eels were the first elopomorph fishes found to contain two copies of the rhodopsin gene in their genomes [19,26]. Recently, the origin of the two copies of *rh1* in the genome of the Japanese eel was investigated, and the authors concluded that these two copies originated in the FSGD event [17,33]. Beyond anguillid eels, we herein identified two copies of *rh1* in most anguilliforms and determined their orthology to either *rh1-dso* (*dso*: “deep-sea” type) or *rh1-fwo* (*fwo*: “freshwater” type) of anguillid eels (Fig 2).

Previous studies showed that anguillid eels adjust their vision to adapt to the photic environment using two copies of the rhodopsin gene (*rh1-dso* and *rh1-fwo*) in different life stages (deep-sea vs. fresh water) [19,26]. Other anguilliform fishes are predominantly benthic marine fishes which do not exhibit a complete catadromous life cycle; they may, however, perform vertical migrations corresponding to different light conditions during their life stages. For example, during the larval or leptocephalus stage, Kaup's cutthroat eels (*Synaphobranchus kaupii*) may stay in the mixed layer shallower than 200 m and then quickly sink to the deep-sea floor (deeper than 1000 m in depth) after metamorphosis from the leptocephalus to the glass eel stage [76]. Maintaining two copies of the rhodopsin gene in the genome might be advantageous for visual adaptation in the evolution of those anguillifom fishes. Conversely, the loss of the duplicated copy in the elopiforms, albuliforms, notacanthiforms, pelican eels, and muranids might be related to a life history in which all life-stages take place in the same environment (only shallow, coastal, or deep-sea).

Yokoyama et al. [21] pinpointed that replacements occurring at 12 key amino acid sites of vertebrate rhodopsin were responsible for the divergence in light absorbance. Those sites are: positions 83, 96, 102, 122, 183, 194, 195, 253, 261, 289, 292, and 317 [21]. By comparing these key amino acid sites from sequences between *rh1-dso* and *rh1-fwo* of each examined species in the Elopomorpha, five of these 12 key sites were observed to exhibit variations including sites 83, 183, 194, 195, and 292 (S4 Table). Beyond those key sites, an additional amino-acid site was found to display a consistent pattern of differences between the two types of rhodopsin (site 210; S4 Table). In previous studies, this amino-acid site was mentioned as exhibiting variable amino acids [cysteine (C)/valine (V)] of rhodopsins throughout the studied taxa (i.e., deep-sea fishes, teleosts, and vertebrates) [14,16,21]. In this study, we found that most “deep-sea” type rhodopsins exhibited V, while “freshwater” types exhibited C for this site. Variations in these six amino acid sites between the compared *rh1-dso* and *rh1-fwo* rhodopsin in each species support the hypothesis of functional divergence between Elops *rh1-dso* and Elops *rh1-fwo*. This hypothesis fits with the mechanism of “subfunctionalization” proposed to explain the long-term maintenance of duplicated genes [40]. This implies that elopomorph fishes might use the two copies of the rhodopsin gene to adjust their light needs during different stages of their life history. However, as previously suggested [6], further investigations, i.e., gene expression and gene functional assessments, are still required to better understand the evolution of opsins in elopomorph fishes and the precise mechanisms of molecular adaptation described above for teleost fishes. Such additional data would eventually highlight the potential consequences of gene duplication in the diversification of elopomorph fishes.

## Supporting information

S1 Fig The gene orthology assessment of the lineage *Albula* spp. *rh1* and *Hiodon alosoides rh1-1*, *rh1-2*. Various possible orthologous relationships were constrained and tested. The result showed that the *Albula* spp. *rh1* is more relative to Elopomorph *rh1-dso* (with higher–ln likelihood value) while the Osteoglossomorph *rh1* is more relative to *Hiodon alosoides rh1-1*.
(TIF)

S2 Fig The reconstruction of ancestral state of *rh1/rh1-like* gene within the jawed vertebrate. The analysis was based on parsimony method. Inferring characters including the presence of the *exo-rh1* (left), intron region of *rh1* (middle), and the number of intron-less *rh1* (right) found in the genome of the organisms.
(TIF)

S3 Fig The schematic rhodopsin gene tree of Lin et al. 2017. Each gene lineage corresponding to gene lineages in this study was indicated in parenthesis.
(TIF)

S1 Table Rhodopsin gene primers used in this study. (DOCX)

S2 Table Samples/sequences used in this study. Voucher number for elopomorph specimens collected in this study was quoted in parenthesis.
(DOCX)

S3 Table Descriptive statistics of each codon of rhodopsin gene sequences. (DOCX)

S4 Table Variable amino acid sites between two types of rhodopsins in the Elopomorpha (Elops *rh1-dso* and *rh1-fwo*). Asterisk indicates the critical sites for functional tuning which were proposed in Yokoyama et al. (2008). Sequences were aligned with the bovine rhodopsin sequence.
(DOCX)
