# Supplementary figures and images for: Rhodopsin gene evolution in early teleost fishes
Source: PLoS One. 2018 Nov 5;13(11):e0206918. doi: 10.1371/journal.pone.0206918 (PMC6218077; doi:10.1371/journal.pone.0206918)

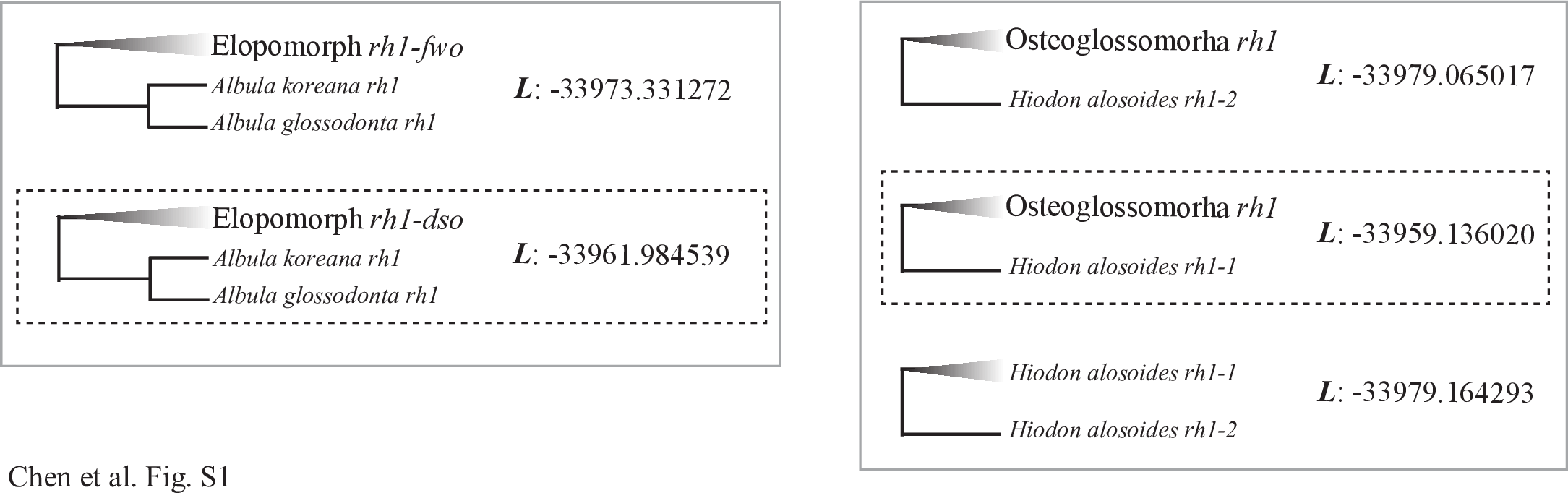

Supplement: S1 Fig — Various possible orthologous relationships were constrained and tested. The result showed that the Albula spp. rh1 is more relative to Elopomorph rh1-dso (with higher–ln likelihood value) while the Osteoglossomorph rh1 is more relative to Hiodon alosoides rh1-1. (TIF) [file pone.0206918.s001.tif]

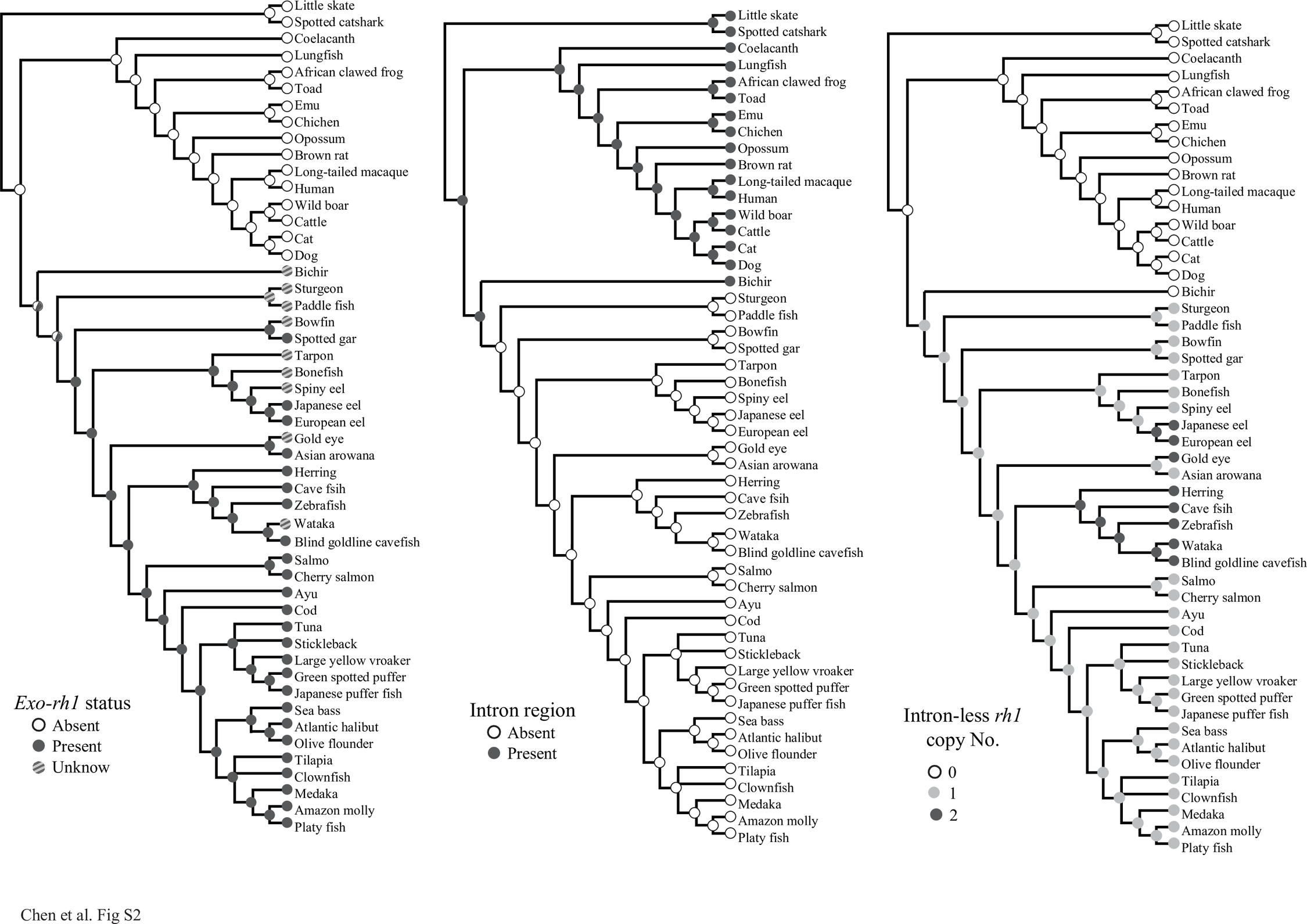

Supplement: S2 Fig — The analysis was based on parsimony method. Inferring characters including the presence of the exo-rh1 (left), intron region of rh1 (middle), and the number of intron-less rh1 (right) found in the genome of the organisms. (TIF) [file pone.0206918.s002.tif]

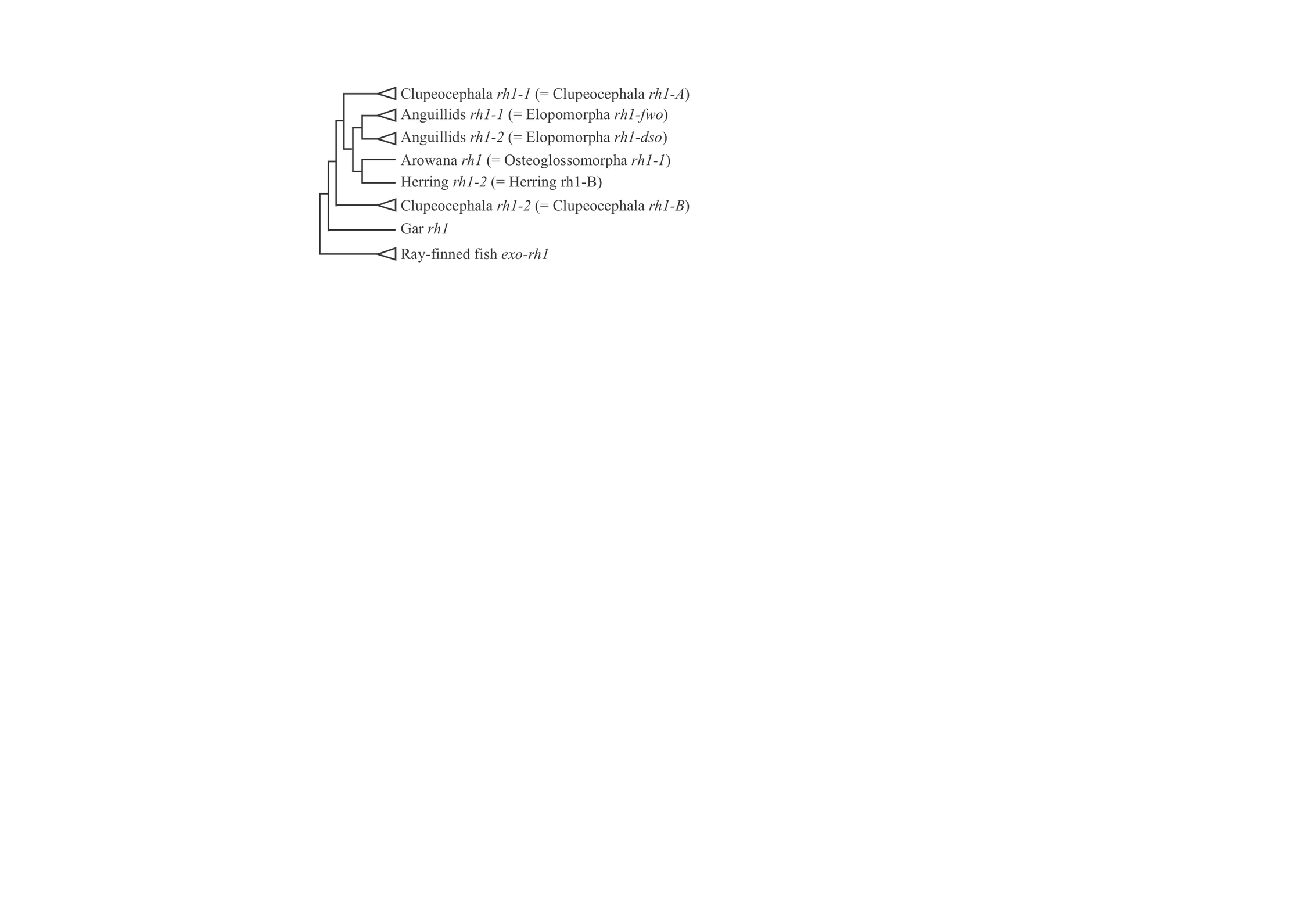

Supplement: S3 Fig — Each gene lineage corresponding to gene lineages in this study was indicated in parenthesis. (TIF) [file pone.0206918.s003.tif]
