# Supplementary material for: Rhodopsin gene evolution in early teleost fishes
Source: PLoS One. 2018 Nov 5;13(11):e0206918. doi: 10.1371/journal.pone.0206918 (PMC6218077; doi:10.1371/journal.pone.0206918)
Supplement: S1 Table — (DOCX) [file pone.0206918.s004.docx]

S1 Table. Rhodopsin gene primers used in this study

| Locus / Primer^1^ | | Primer sequences (5'-3') | Source |
| --- | --- | --- | --- |
|  | RH_1F^3^ | ATGAACGGCACAGARGGAC | Chen et al. (2003) |
|  | RH_1F_ds^2^  RH_47F | ATGAACGGCRCAGAGGGC  CCACCGGCATNGTGAGRAG | This study  This study |
|  | RH_193F  RH_CharaF^5^  RH_HiodonF^4^ | CNTATGAATAYCCTCAGTACTACC  AGCTTTTGGCAAARAANGAGG  CATGGATGTACTCGGTGCTG | Chen et al. (2003)  This study  This study |
|  | *RH_997R*^2^  *RH_1010R^4^* | *GAGCTCTTGGCAAAGAAGGAG*  *TTGTAGATGGCCGAGCTCTT* | This study  This study |
|  | *RH_1039R* | *TGCTTGTTCATGCAGATGTAGA* | Chen et al. (2003) |
|  | *RH_1073R*^3^  *RH_CharaR*^5^ | *CCRCAGCACAGRGTGGTGATCATG*  *GATCGCTGCATACATGTTSTT* | Chen et al. (2003)  This study |

^1^ Reverse primers indicated in Italics.

^2^ Primer pair used to amplify and sequence the fragment of deep-sea type rhodopsin gene in the Elopomorpha.

^3^ Primer pair used to amplify and sequence the fragment of Osteoglossomorph *rh1-2* of *Hiodon alosoides*.

^4^ Primer pair used to amplify and sequence the fragment of Osteoglossomorph *rh1-1* of *Hiodon alosoides*

^5^ Primer pair used to amplify and sequence the fragment of rhodopsin gene copy (*rh1-B*) in the Otocephala.

Reference

Chen W-J, Bonillo C, Lecointre G. Repeatability of clades as a criterion of reliability: a case study for molecular phylogeny of Acanthomorpha (Teleostei) with larger number of taxa. Mol Phylogenet Evol. 2003;26: 262-288.
