# Supplementary material for: Rhodopsin gene evolution in early teleost fishes
Source: PLoS One. 2018 Nov 5;13(11):e0206918. doi: 10.1371/journal.pone.0206918 (PMC6218077; doi:10.1371/journal.pone.0206918)
Supplement: S2 Table — Voucher number for elopomorph specimens collected in this study was quoted in parenthesis. (DOCX) [file pone.0206918.s005.docx]

**S2 Table. Samples/sequences used in this study.** Voucher number for elopomorph specimens collected in this study was quoted in parenthesis.

|  |  | Taxon | GenBank accession no. | | |
| --- | --- | --- | --- | --- | --- |
|  |  | Species (voucher no.) | ***rh1*** |  | ***exo-rh1*** |
| Chondrichthyes | Skate | *Raja erinacea* | U81514 |  |  |
|  | Shark | *Scyliorhinus canicula* | Y17585 |  |  |
| Sarcopterygii | Coelacanth | *Latimeria chalumnae* | AH007712 |  |  |
|  | Lungfish | *Protopterus* sp. | AF369054 |  |  |
|  | Tetrapod | *Bufo marinus* | U59922 |  |  |
|  |  | *Xenopus laevis* | U23808 |  |  |
|  |  | *Gallus gallus* | D00702 |  |  |
|  |  | *Dromaius novaehollandiae* | KU568456 |  |  |
|  |  | *Caluromys philander* | AY313946 |  |  |
|  |  | *Rattus norvegicus* | NM033441 |  |  |
|  |  | *Bos taurus* | M21606 |  |  |
|  |  | *Sus scrofa* | AF008947 |  |  |
|  |  | *Canis familiaris* | AY092841 |  |  |
|  |  | *Felis catus* | AJ417432 |  |  |
|  |  | *Macaca fascicularis* | S76579 |  |  |
|  |  | *Homo sapiens* | NM000539 |  |  |

| Actinopterygii |  | *Acipenser* sp. | AF137206 |  |  |
| --- | --- | --- | --- | --- | --- |
|  |  | *Polyodon spathula* | AF369050 |  |  |
|  |  | *Lepisosteus oculatus* | Ensembl (LOC102698275) |  | Ensembl |
|  |  | *Lepisosteus osseus* | AF137207 |  |  |
|  |  | *Amia calva* | AF137208 |  |  |

|  |  | Taxon | GenBank accession no. | | | | | |
| --- | --- | --- | --- | --- | --- | --- | --- | --- |
|  |  | Species (voucher no.) | ***rh1-dso rh1-fwo*** | | |  | ***exo-rh1*** | |
| Teleostei | Elopomorpha | *Elops saurus* (non-catalogued) ^g^ | MH796447 |  | | | |  |
|  | (eel and relatives) | *Megalops atlanticus* (non-catalogued) ^g^ | MH796448 |  | | | |  |
|  |  | *Megalops cyprinoides* (NTUM12845) ^f^ | MH796449 |  | | | |  |
|  |  | *Albula koreana* (NTUM12807) ^f^ | MH796450 |  | | | |  |
|  |  | *Albula glossodonta* (non-catalogued) ^f^ | MH796451 |  | | | |  |
|  |  | *Halosaurus ridgwayi* (non-catalogued) ^a^ | MH796452 |  | | | |  |
|  |  | *Aldrovandia affinis* (non-catalogued) ^a^ | MH796453 |  | | | |  |
|  |  | *Notacanthus abbotti* (NTUM12859) ^f^ | MH796454 |  | | | |  |
|  |  | *Notacanthus bonaparte* | JN544543 |  | | | |  |
|  |  | *Protanguilla palau* (non-catalogued) ^g^ | MH796455 | MH796494 | | | |  |
|  |  | *Dysomma anguillare* (NTUM12866) ^f^ | MH796456 | MH764300 | | | |  |
|  |  | *Meadia abyssalis* (non-catalogued) ^g^ |  | MH796495 | | | |  |
|  |  | *Synaphobranchus affinis* (NTUM12865) ^b^ | MH796457 | MH796496 | | | |  |
|  |  | *Synaphobranchus kaupii* (NTUM14257) ^d^ | MH796458 |  | | | |  |
|  |  | *Histiobranchus bathybius* | JN544542 |  | | | |  |
|  |  | *Gymnothorax buroensis* (NTUM10047) ^b^ |  | MH796497 | | | |  |
|  |  | *Gymnothorax favagineus* |  | HQ444181 | | | |  |
|  |  | *Gymnothorax minor* (NTUM12849) ^f^ |  | MH796498 | | | |  |
|  |  | *Gymnothorax tile* (non-catalogued) ^f^ |  | MH796499 | | | |  |
|  |  | *Monopenchelys acuta* (NTUM10299) ^b^ |  | MH796500 | | | |  |
|  |  | *Rhinomuraena quaesita* |  | HQ444180 | | | |  |
|  |  | *Strophidon sathete* |  | HQ444183 | | | |  |
|  |  | *Uropterygius fasciolatus* (NTUM10222) ^b^ |  | MH796501 | | | |  |
|  |  | *Anguilla anguilla* | L78008 | L78007 | | | | Ensembl |
|  | |  |  |  | | | |  |
|  |  | Taxon | GenBank accession no. | | | | | |
|  |  | Species (voucher no.) | ***rh1-dso rh1-fwo*** | |  | | ***exo-rh1*** | |
|  |  | *Anguilla japonica* | AJ249203 | AJ249202 | | | | Ensembl |
|  |  | *Anguilla marmorata* | KJ462781 | KJ462782 | | | |  |
|  |  | *Moringua macrocephalus* (NTUM12887) ^f^ | MH796459 | MH796502 | | | |  |
|  |  | *Moringua microchir* (NTUM10217) ^b^ | MH796460 | MH796503 | | | |  |
|  |  | *Nemichthys scolopaceus* (NTUM12852) ^f^ | MH796461 | MH796504 | | | |  |
|  |  | *Serrivomer lanceolatoides* (NTUM12888) ^c^ | MH796462 | MH796505 | | | |  |
|  |  | *Serrivomer sector* (KU 27965) ^g^ |  | MH796506 | | | |  |
|  |  | *Eurypharynx pelecanoides* (ASIZP0067331) ^g^ |  | MH796507 | | | |  |
|  |  | *Kaupichthys* sp. (NTUM10220) ^b^ | MH796463 | MH796508 | | | |  |
|  |  | *Chlopsis slusserorum* (NTUM12871) ^b^ | MH796464 | MH796509 | | | |  |
|  |  | *Coloconger scholesi* (NTUM14251) ^b^ | MH796465 | MH796510 | | | |  |
|  |  | *Coloconger reniceps* (non-catalogued) ^d^ | MH796466 | MH796511 | | | |  |
|  |  | *Congriscus megastomus* (NTUM13331) ^e^ | MH796467 | MH796512 | | | |  |
|  |  | *Congriscus maldivensis* (NTUM13792) ^e^ | MH796468 | MH796513 | | | |  |
|  |  | *Ariosoma major* (NTUM12815) ^f^ | MH796469 | MH796514 | | | |  |
|  |  | *Bathycongrus retrotinctus* (NTUM12810) ^f^ | MH796470 | MH796515 | | | |  |
|  |  | *Bathyuroconger parvibranchialis* (NTUM12812) ^f^ | MH796471 | MH796516 | | | |  |
|  |  | *Conger conger* | MH796472 | MH796517 | | | |  |
|  |  | *Conger japonicus* (NTUM12811) ^f^ | MH796473 | MH796518 | | | |  |
|  |  | *Conger myriaster* | AB043818 | AB043817 | | | |  |
|  |  | *Gnathophis heterognathos* (NTUM12814) ^f^ | MH796474 | MH796519 | | | |  |
|  |  | *Gnathophis* sp*.* (NTUM13344) ^f^ | MH796475 | MH796520 | | | |  |
|  |  | *Macrocephenchelys brachialis* (NTUM12813) ^f^ | MH796476 | MH796521 | | | |  |
|  |  | Taxon | GenBank accession no. | | | | | |
|  |  | Species (voucher no.) | ***rh1-dso rh1-fwo*** | | |  | ***exo-rh1*** | |
|  |  | *Macrocephenchelys soela* (NTUM11861) ^b^ | MH796477 | MH796522 | | | |  |
|  |  | *Parabathymyrus macrophthalmus* (NTUM12818) ^f^ | MH796478 | MH796523 | | | |  |
|  |  | *Rhynchoconger ectenurus* (NTUM12817) ^f^ | MH796479 | MH796524 | | | |  |
|  |  | *Nessorhamphus danae* (NTUM12119) ^b^ | MH796480 | MH796525 | | | |  |
|  |  | *Derichthys serpentinus* (MCZ 159068) ^g^ |  | MH796526 | | | |  |
|  |  | *Gavialiceps taiwanensis*  (NTUM12846 for *rh1-dso*), (NTUM12884 for *rh1-fwo*) ^f^ | MH796481 | MH796527 | | | |  |
|  |  | *Muraenesox cinereus* (NTUM12848) ^f^ | MH796482 | MH796528 | | | |  |
|  |  | *Oxyconger leptognathus* (NTUM13343) ^f^ | MH796483 | MH796529 | | | |  |
|  |  | *Facciolella equatorialis* (SIO No. 95-2) ^g^ | MH796484 | MH796530 | | | |  |
|  |  | *Nettastoma solitarium* (ASIZP0911532) ^g^ | MH796485 |  | | | |  |
|  |  | *Venefica tentaculate* (NTUM12858) ^e^ | MH796486 | MH796531 | | | |  |
|  |  | *Apterichtus klazingai* (NTUM10013) ^b^ | MH796487 | MH796532 | | | |  |
|  |  | *Brachysomophis henshawi* (NTUM10268) ^b^ | MH796488 | MH796533 | | | |  |
|  |  | *Ophichthus machidai* (NTUM12860) ^f^ | MH796489 | MH796534 | | | |  |
|  |  | *Neenchelys* sp. (non-catalogued) ^f^ | MH796490 | MH796535 | | | |  |
|  |  | *Muraenichthys johnstonensis* (non-catalogued) ^f^ | MH796491 | MH796536 | | | |  |
|  |  | *Myrophis microchir* (NTUM13321) ^b^ | MH796492 | MH796537 | | | |  |
|  |  | *Yirrkala* sp. (non-catalogued) ^g^ | MH796493 | MH796538 | | | |  |
|  |  |  | ***rh1-1*** | ***rh1-2*** | | | |  |
|  | Osteoglossomorpha | *Hiodon alosoides* | MH796542 | MH796543 | | | |  |
|  | (bony tongue) | *Pantodon buchholzi* | AF137210 |  | | | |  |
|  |  | *Arapaima gigas* | JN230972 |  | | | |  |
|  |  | *Mormyrops anguilloides* | JN230973 |  | | | |  |
|  |  | *Scleropages formosus* | LGSG01034095 |  | | | | Ensembl |
|  |  | Taxon | GenBank accession no. | | | | | |
|  |  | Species (voucher no.) | ***rh1-A rh1-B*** | | |  | ***exo-rh1*** | |
|  | Otocephala | *Alepocephalus bicolor* | JN230974 |  | | | |  |
|  |  | *Clupea harengus* | JZKK01059327 | JZKK01032363 | | | | shotgun sequences |
|  |  | *Denticeps clupeoides* | JN230976 |  | | | |  |
|  |  | *Chanos chanos* | JN230981 |  | | | |  |
|  |  | *Apteronotus albifrons* | JN230983 |  | | | |  |
|  |  | *Astyanax mexicanus* | KB882218 | MH796540 | | | | XM_007238280 |
|  |  | *Ctenolucius hujeta* | JN230987 | MH796541 | | | |  |
|  |  | *Pygocentrus nattereri* | MAUM01028547 |  | | | |  |
|  |  | *Corydoras rabauti* | FJ197074 |  | | | |  |
|  |  | *Ictalurus punctatus* | LBML01009420 |  | | | |  |
|  |  | *Carassius auratus* | L11863 | KY026043 | | | |  |
|  |  | *Cyprinus carpio* | LHQP01039061 |  | | | | XM_019093139 |
|  |  | *Danio rerio* | Ensembl (Chr7) | Ensembl (Chr11) | | | | Ensembl (Chr8) |
|  |  | *Epalzeorhynchos bicolor* | HQ286332 | HQ286329 | | | |  |
|  |  | *Ischikauia steenackeri* | EU409648 | MH796539 | | | |  |
|  |  | *Pimephales promelas* | JNCE01103361 |  | | | |  |
|  |  | *Sinocyclochelius anshuiensis* | LAVE01001134 | LOC107696845 | | | | XM_016499451 |
|  | Euteleostei | *Salmon salar* | LOC100136370 |  | | | | NM_001123536 |
|  |  | *Oncorhynchus masou* | AB772406 |  | | | | AB772407 |
|  |  | *Esox americanus* | JN230998 |  | | | |  |
|  |  | *Umbra limi* | JN230999 |  | | | |  |
|  |  | *Argentina sialis* | JN230995 |  | | | |  |
|  |  | Taxon | GenBank accession no. | | | | | |
|  |  | Species (voucher no.) | ***rh1-A rh1-B*** | | |  | ***exo-rh1*** | |
|  |  | *Bathylagus euryops* | AY141255 |  | | | |  |
|  |  | *Galaxias maculatus* | JN231000 |  | | | |  |
|  |  | *Lepidogalaxias salamandroides* | JX255565 |  | | | |  |
|  |  | *Osmerus eperlanus* | JN230996 |  | | | |  |
|  |  | *Plecoglossus altivelis* | AB086404 |  | | | | AB089247 |
|  |  | *Polyipnus stereope* | JN230997 |  | | | |  |
|  |  | *Bathypterois dubius* | AY141257 |  | | | |  |
|  |  | *Bathysaurus ferox* | JN412585* |  | | | |  |
|  |  | *Bathysaurus mollis* | JN412586* |  | | | |  |
|  |  | *Chlorophthalmus acutifrons* | KC442222 |  | | | |  |
|  |  | *Harpadon microchir* | KC442220 |  | | | |  |
|  |  | *Hime japonica* | KC442221 |  | | | |  |
|  |  | *Lestrolepis japonica* | KC442223 |  | | | |  |
|  |  | *Saurida elongata* | KC442219 |  | | | |  |
|  |  | *Scopelarchus analis* | EF517404/ EF517405 |  | | | |  |
|  |  | *Synodus foetens* | JN231001 |  | | | |  |
|  |  | *Diaphus watasei* | JN231003 |  | | | |  |
|  |  | *Gadus morhua* | Ensembl (Scaffold_4578) |  | | | | Ensembl (Scaffold_745) |
|  |  | *Merluccius merluccius* | JN231004 |  | | | |  |
|  |  | *Beryx splendens* | AY141265 |  | | | |  |
|  |  | *Anoplopoma fimbria* | AWGY01146807 |  | | | |  |
|  |  | *Cottus rhenanus* | LKTN01144029 |  | | | |  |
|  |  | Taxon | GenBank accession no. | | | | | |
|  |  | Species (voucher no.) | ***rh1-A rh1-B*** | | |  | ***exo-rh1*** | |
|  |  | *Gasterosteus aculeatus* | KC774627 |  | | | | Ensembl (groupXII) |
|  |  | *Cyprinodon nevadensis* | JSUU01011719 |  | | | |  |
|  |  | *Poecilia formosa* | KI519745 |  | | | | Ensembl |
|  |  | *Xiphophorus maculatus* | JH556804 |  | | | | JH556804 |
|  |  | *Amphiprion melanopus* | HM107824 |  | | | | HM107820 |
|  |  | *Astronotus ocellatus* | EF095629 |  | | | |  |
|  |  | *Oreochromis niloticus* | GL831137 |  | | | | Ensembl |
|  |  | *Thunnus orientalis* | AB290449 |  | | | | BADN01008200 |
|  |  | *Larimichthys_crocea* | KP723006 |  | | | | XM_010756793 |
|  |  | *Lates calcarifer* | AY141294 |  | | | |  |
|  |  | *Paralichthys olivaceus* | KC442236 |  | | | | HM107825 |
|  |  | *Hippoglossus hippoglossus* | AF156265 |  | | | | KF941294 |
|  |  | *Oryzias latipes* | Ensembl (Chr7) |  | | | | Ensembl |
|  |  | *Takifugu rubripes* | scaffold_527 |  | | | | Ensembl |
|  |  | *Tetraodon nigroviridis* | Ensembl (Chr9) |  | | | | Ensembl |

^a^ National Taiwan University Museums (NTUM) specimens collected through the Tropical Deep-Sea Benthos (TDSB) program (campaign: EXBODI) and used to obtain the new sequences in this study.

^b^ NTUM specimens collected through the TDSB (campaign: PAPUA NIUGINI) and used to obtain the new sequences in this study.

^c^ NTUM specimens collected through the TDSB (campaign: Taiwan 2013) and used to obtain the new sequences in this study.

^d^ NTUM specimens collected through the TDSB (campaign: NanHai 2014) and used to obtain the new sequences in this study.

^e^ NTUM specimens collected through the TDSB (campaign: Zhongsha 2015) and used to obtain the new sequences in this study.

^f^ NTUM specimens collected from Da-shi and Dongang and used to obtain the new sequences in this study.

^g^ Tissue samples provided by collaborators including Drs. K.-C. Shao, H.-C. Ho and C.-H. Chang, and other organizations including Scrips Institution of Oceanography (SIO), KU Biodiversity institute & Natural History Museum, Cryobanking Program for wildlife Genetic Material in Taiwan managed by Academic Sinica (ASIZP).
