# Supplementary material for: Rhodopsin gene evolution in early teleost fishes
Source: PLoS One. 2018 Nov 5;13(11):e0206918. doi: 10.1371/journal.pone.0206918 (PMC6218077; doi:10.1371/journal.pone.0206918)
Supplement: S3 Table — (DOCX) [file pone.0206918.s006.docx]

**S3 Table. Descriptive statistics of each codon of rhodopsin gene sequences.**

| Codon | 1 | 2 | 3 |
| --- | --- | --- | --- |
| Length (base pair) | 332 | 332 | 332 |
| No. of variable sites (in %) | 230 (58%) | 199 (60%) | 323 (97%) |
| No. of parsimony-informative sites | 192 | 140 | 318 |
| Base frequency homogenity (p)^a^ | 1 | 1 | 0* |

a. *P* value from Chi-square test of homogeneity of base frequencies across taxa.

* Asterisk sign indicates that the data is significantly rejected by Chi-square test.
