# Supplementary material for: Rhodopsin gene evolution in early teleost fishes
Source: PLoS One. 2018 Nov 5;13(11):e0206918. doi: 10.1371/journal.pone.0206918 (PMC6218077; doi:10.1371/journal.pone.0206918)
Supplement: S4 Table — Asterisk indicates the critical sites for functional tuning which were proposed in Yokoyama et al. (2008). Sequences were aligned with the bovine rhodopsin sequence. (DOCX) [file pone.0206918.s007.docx]

**S4 Table. Variable amino acid sites between two types of rhodopsins in the Elopomorpha (gene: Elops *rh1-dso* and *rh1-fwo*)**. Asterisk indicates the critical sites for functional tuning which were proposed in Yokoyana et al. [22]. Sequences have been aligned with the bovine rhodopsin sequence.

| **Sequence** | **Amino acid site** | | | | | |
| --- | --- | --- | --- | --- | --- | --- |
|  | 83* | 183* | 194* | 195* | 210 | 292* |
| *Elops saurus rh1* | D | M | L | K | V | A |
| *Megalops atlanticus rh1* | D | M | L | K | V | - |
| *Megalops cyprinoides rh1* | D | M | L | K | V | A |
| *Notacanthus abbotti rh1* | N | L | L | K | V | S |
| *Aldrovandia affinis rh1* | - | L | L | K | V | - |
| *Halosaurus ridgwayi rh1* | N | L | L | K | V | S |
| *Notacanthus bathybius rh1* | N | L | L | K | V | S |
| *Dysomma anguillare rh1-dso* | N | M | L | S | V | S |
| *Protoanguilla palau rh1-dso* | N | L | L | S | V | - |
| *Synaphobranchus kaupii rh1-dso* | N | L | L | S | V | S |
| *Histiobranchus bathybius rh1-dso* | N | M | L | N | V | S |
| *Synaphobranchus affinis rh1-dso* | N | L | L | N | V | S |
| *Moringua macrocephalus rh1-dso* | N | M | L | K | V | S |
| *Moringua microchir rh1-dso* | N | L | L | K | V | S |
| *Chlopsis slusserorum rh1-dso* | N | L | L | K | V | S |
| *Kaupichthys* sp. *rh1-dso* | N | L | L | K | V | S |
| *Nemichthys scolopaceus rh1-dso* | D | M | L | N | V | S |
| *Serrivomer lanceolatoides rh1-dso* | N | M | L | H | V | S |
| *Anguilla japonica rh1-dso* | N | M | L | K | V | S |
| *Anguilla marmorata rh1-dso* | N | M | L | K | V | S |
| *Anguilla anguilla rh1-dso* | N | M | L | K | V | S |
| *Nessorhamphus danae rh1-ds* | N | L | L | K | V | S |
| *Coloconger scholesi rh1-dso* | N | L | L | K | V | S |
| *Coloconger reniceps rh1-dso* | N | L | L | K | V | S |
| *Congriscus megastomus rh1-dso* | N | L | R | K | V | S |
| *Congriscus maldivensis rh1-dso* | N | L | R | K | V | S |
| *Muraenesox cinereus rh1-dso* | D | L | L | K | V | S |
| *Apterichtus klazingai rh1-dso* | D | L | L | K | V | - |
| *Yirrkala misolensis rh1-dso* | D | L | L | K | V | S |
| *Ophichthus machidai rh1-dso* | N | L | L | K | V | - |
| *Brachysomophis henshawi rh1-dso* | D | L | L | K | V | S |
| *Muraenichthys jhonstonesis rh1-dso* | N | L | L | K | V | - |
| *Myrophis microchir rh1-dso* | N | L | L | K | V | S |
| *Neenchelys* sp. *rh1*-*dso* | N | L | L | K | V | S |
| *Oxyconger leptocegnathus rh1-dso* | N | L | L | K | V | S |
| **Sequence** | **Amino acid site** | | | | | |
|  | 83* | 183* | 194* | 195* | 210 | 292* |
| *Ariosoma major rh1-dso* | D | L | L | K | V | S |
| *Parabathymyrus macrophthalmus rh1-dso* | N | L | L | K | V | - |
| *Rhynchoconger ectenurus rh1-dso* | N | L | L | K | V | S |
| *Conger myriaster rh1-dso* | D | L | L | K | V | S |
| *Conger conger rh1-dso* | D | L | L | K | V | S |
| *Conger cinereus rh1-dso* | D | M | R | K | V | S |
| *Conger japonicus rh1-dso* | D | M | L | K | V | S |
| *Facciolella equatorialis rh1-dso* | D | L | L | K | V | - |
| *Nettastoma solitarium rh1-dso* | N | M | L | K | V | - |
| *Venefica tentaculata rh1-dso* | N | L | L | K | V | S |
| *Gavialiceps taiwanensis rh1-dso* | N | M | L | K | V | S |
| *Bathyuroconger parvibranchialis rh1-dso* | N | L | L | K | V | S |
| *Bathycongrus retrotinctus rh1-dso* | N | M | L | K | V | - |
| *Bathycongrus wallacei rh1-dso* | N | M | L | K | V | S |
| *Gnathophis* sp. *rh1-dso* | D | L | L | K | C | S |
| *Gnathophis heterognathos rh1-dso* | D | L | L | K | C | S |
| *Macrocephenchelys brachialis rh1-dso* | N | L | L | K | V | S |
| *Macrocephenchelys solea rh1-dso* | N | L | L | K | V | S |
| *Albula glossodonta rh1* | D | M | K | N | C | A |
| *Albula koreana rh1* | D | L | K | N | C | A |
| *Gnathophis heterognathos rh1-fwo* | N | L | R | S | C | A |
| *Gnathophis* sp*. rh1-fwo* | N | L | R | S | C | A |
| *Uropterygius fasciolatus rh1-fwo* | D | L | R | A | C | S |
| *Monopenchelys acuta rh1-fwo* | D | L | R | A | C | S |
| *Gymnothorax favagineus rh1-fwo* | D | L | R | A | C | S |
| *Gymnothorax buroensis rh1-fwo* | D | L | R | A | C | S |
| *Gymnothorax tile rh1-fwo* | D | L | R | A | C | S |
| *Rhinomuraena quaesita rh1-fwo* | D | L | R | A | C | A |
| *Gymnothorax minor rh1-fwo* | D | L | R | A | C | S |
| *Strophidon sathete rh1-fwo* | D | L | R | A | - | S |
| *Bathyuroconger parvibranchialis rh1-fwo* | N | L | R | S | C | S |
| *Bathycongrus wallacei rh1-fwo* | N | L | R | S | C | S |
| *Bathycongrus retrotinctus rh1-fwo* | N | L | R | S | C | S |
| *Protoanguilla palau rh1-fwo* | N | M | R | S | C | S |
| *Synaphobranchus affinis rh1-fwo* | N | M | R | S | C | A |
| *Meadia abyssalis rh1-fwo* | N | M | R | S | C | S |
| *Dysomma anguillare rh1-fwo pseudo* | N | - | R | S | C | S |
| *Chlopsis slusserorum rh1-fwo* | N | L | M | A | C | S |
| **Sequence** | **Amino acid site** | | | | | |
|  | 83* | 183* | 194* | 195* | 210 | 292* |
| *Kaupichthys* sp. *rh1-fwo* | N | L | R | S | C | S |
| *Coloconger scholesi rh1-fwo* | D | L | R | S | C | S |
| *Coloconger reniceps rh1-fwo* | D | L | R | S | C | I |
| *Coloconger scholesi rh1-fwo* | D | L | R | S | C | S |
| *Nessorhamphus danae rh1-fwo* | D | L | R | S | C | - |
| *Derichthys serpentinus rh1-fwo* | D | L | R | A | V | S |
| *Congriscus megastomus rh1-fwo* | N | L | L | S | C | S |
| *Congriscus maldivensis rh1-fwo* | N | L | L | S | C | S |
| *Moringua microchir rh1-fwo* | D | L | R | S | C | S |
| *Moringua macrocephalus rh1-fwo* | D | L | R | S | C | S |
| *Eurypharynx pelecanoides rh1* | D | M | L | N | C | S |
| *Nemichthys scolopaceus rh1-fwo* | N | I | P | N | C | A |
| *Serrivomer sector rh1-fwo* | D | M | L | N | - | A |
| *Serrivomer lanceolatoides rh1-fwo* | D | M | L | N | L | A |
| *Anguilla anguilla rh1-fwo* | D | M | P | N | C | A |
| *Anguilla marmorata rh1-fwo* | D | M | P | N | C | A |
| *Anguilla aponica rh1-fwo* | D | M | P | N | C | A |
| *Conger myriaster rh1-fwo* | D | L | R | A | C | S |
| *Conger conger rh1-fwo* | N | L | R | A | C | S |
| *Conger cinerus rh1-fwo* | N | L | L | A | C | S |
| *Conger japonicus rh1-fwo* | N | L | R | A | C | S |
| *Venefica tentaculata rh1-fwo* | N | L | R | S | C | S |
| *Macrocephenchelys solea rh1-fwo* | N | L | R | S | C | S |
| *Macrocephenchelys brachialis rh1-fwo* | N | L | R | S | C | S |
| *Rhynchoconger ectenurus rh1-fwo* | D | L | R | S | C | S |
| *Facciolella equatorialis rh1-fwo* | N | L | R | S | C | - |
| *Gavialiceps taiwanensis rh1-fwo* | D | L | R | S | C | S |
| *Oxyconger leptocegnathus rh1-fwo* | D | L | R | S | C | A |
| *Muraenesox cinereus rh1-fwo* | D | L | R | S | C | A |
| *Ariosoma major rh1-fwo* | N | L | L | S | C | A |
| *Ariosoma meeki rh1-fwo* | N | L | L | S | C | A |
| *Parabathymyrus macrophthalmus rh1-fwo* | N | L | L | K | V | - |
| *Neenchelys* sp*. rh1-fwo* | D | L | R | S | C | A |
| *Muraenichthys johnstonesis rh1-fwo* | D | L | R | S | C | A |
| *Myrophis microchir rh1-fwo* | D | L | R | S | C | S |
| *Yirrakala misolensis rh1-fwo* | D | L | R | S | C | A |
| *Apterichtus klazingai rh1-fwo* | D | L | R | S | C | A |
| *Ophichthus machidai rh1-fwo* | - | L | R | S | C | A |
| **Sequence** | **Amino acid site** | | | | | |
|  | 83* | 183* | 194* | 195* | 210 | 292* |
| *Brachysomophis henshawi rh1-fwo* | N | L | R | S | C | A |
